# Balance Level and Fundamental Motor Skills of Youth with Visual Impairments: Pilot Study

**DOI:** 10.3390/jcm14103483

**Published:** 2025-05-16

**Authors:** Grzegorz Bednarczuk, Wiktoria Bandura, Izabela Rutkowska, Michal Starczewski

**Affiliations:** Faculty of Rehabilitation, Józef Piłsudski University of Physical Education, 00-968 Warsaw, Poland; grzegorz.bednarczuk@awf.edu.pl (G.B.); izabela.rutkowska@awf.edu.pl (I.R.); michal.starczewski@awf.edu.pl (M.S.)

**Keywords:** fundamental motor skills, balance, locomotion, visual impairment, physical activity

## Abstract

**Background:** Visual impairment significantly affects balance and motor skills in children, often leading to postural instability and locomotor difficulties, thereby affecting lifestyle and general health. The aim of this study was to assess balance level and fundamental motor skills in the locomotion of youth with regard to their level of visual impairment. **Methods:** The pilot study included 25 physically active young people with visual impairments, divided into three groups (B1, B2, B3) based on the severity of impairment. Balance was assessed using the AMTI AccuSway platform, both with eyes open and eyes closed. Locomotor skills were evaluated using the TGMD-3. Statistical analysis involved Kruskal–Wallis, ANOVA, and correlation tests. **Results:** Significant differences in balance were found between the B1 and B2 groups, with poorer balance in individuals with more severe visual impairments, particularly in static conditions. No significant differences in locomotor skills were observed between groups. However, girls performed better in balance tests, particularly with eyes closed. A positive correlation was found between balance and locomotor skills in the B2 and B3 groups. **Conclusions:** Visual impairment negatively impacts balance, particularly in individuals with more severe impairments. However, no significant differences were found in locomotor skills between the groups. Regular physical activity supports motor development. Targeted interventions are necessary to improve balance and locomotor skills, especially in children with more severe visual impairments.

## 1. Introduction

Vision is a key sense in assessing body positioning and enabling posture adjustments. Sensory input helps create a reference frame for detecting body sway and initiating corrective responses [1,2,3]. Visual perception plays a crucial role in movement by enabling spatial orientation, avoiding obstacles, adapting to various surfaces, and accurately localizing objects. It enhances spatial awareness and the detection of dynamic elements in the environment, which is particularly important for balance-related motor control. Maintaining balance is a complex process that relies on predictive postural adjustments, coordinated through the integration of the visual, vestibular, and somatosensory systems [4,5,6,7,8].

The severity of visual impairment significantly affects the pace of motor development, with more profound deficits observed in children who are congenitally blind [9,10,11,12]. Early limitations in intersensory coordination can hinder the acquisition of fundamental motor skills, particularly those related to locomotion [6]. Motor development encompasses both qualitative and quantitative changes [13] and follows a systematic process, where visual perception is closely linked to learning new movements. Key factors shaping early motor abilities include the child’s activity level, posture, balance, visual orientation, mobility, and manual skills [14]. Unlike structured instructions, infants develop motor skills through natural interaction with their environment, where the quality of engagement between the individual, task, and surroundings plays a pivotal role [15]. In the absence of visual input, blind children often experience delayed locomotor development due to reduced environmental stimulation and an inability to observe and imitate peers, which may also lead to the formation of incorrect motor patterns [10,11,12,16,17,18]. As a fundamental form of movement, running is often underdeveloped in blind children, who may instead adopt fast walking due to balance challenges [12,19,20].

Research consistently highlights the positive link between physical activity and motor competence, particularly in children with varying degrees of visual impairment. Regrettably, children and adolescents with visual impairment have limited opportunities to participate in various physical activities. This leads to lower than recommended physical activity levels. The less serious the impairment, the more time children spend on physical activity. Children with serious impairment spend more time on activities in sitting positions [21]. The level of motor competence is directly related to the degree of impairment and level of activity [22]. Fundamental motor skills developed in early childhood provide a basis for more advanced physical and sports-related activities. Conversely, children with lower motor competence may show reluctance toward physical activity, which can lead to a sedentary lifestyle in the future [23]. Physical education should play an essential role in encouraging motor skills, which are considered to be the basis for building motor experience. Its development is essential for success, both in organized and in spontaneous forms of activity or sports. Fundamental motor skills are considered to be the basis for the acquisition of more advanced forms of activities, as well as particular sports skills [24,25]. This means that parents, and later sports teachers, are obliged to expose children to rich environmental contexts so that a child with a visual impairment can learn basic motor skills [15]. To increase the involvement of children with visual impairments in sports and recreation, it is necessary to help them develop motor abilities, especially those based on closed chains. The support of instructors, parents, and teachers in learning suitable skills may increase the participation of children with visual impairments in various physical activities [21].

It is noteworthy that limited fundamental motor abilities in children with visual impairments—compared to their sighted peers—can reduce their engagement in health-promoting activities, such as regular physical exercise. This may result in an increased risk of developing numerous metabolic diseases, such as type 2 diabetes [26]. Although visually impaired children may follow a different developmental path, with appropriate strategies they can reach similar milestones as their sighted peers [12]. The use of tests, such as the Test of Gross Motor Development (TGMD), may provide detailed information on exact areas for improvement [21]. The TGMD may be applied for various aims, including the diagnosis of delays in motor ability development, the assessment of progress, and as a measurement tool for studies on motor development [27].

While previous studies have demonstrated that visual impairment affects both balance and gross motor performance, most have either focused on one domain or did not differentiate the results by the severity of visual impairment. For example, Haibach et al. (2014) [28] and Wagner et al. (2013) [23] assessed motor skills using the TGMD-2 without examining the interaction with balance or functional VI classification. Other studies [29,30] primarily evaluated the psychometric properties of the TGMD or its general applicability in children with disabilities. There remains a lack of studies examining both balance and locomotor skills simultaneously in youth with visual impairments and using the updated TGMD-3 tool. Therefore, the present study aims to assess the relationship between balance and fundamental locomotor skills in physically active youth with visual impairment, considering the degree of impairment.

The conducted literature review reveals that the consequences of visual impairment in the context of motor development issues resulting from balance disorders may have a significant impact on the lifestyle and quality of life, as well as on the general health, of those affected. Therefore, the aim of this study was to assess the balance level and fundamental motor skills in the locomotion of youth with regard to their level of visual impairment.

## 2. Material and Methods

### 2.1. Participants

The study group consisted of 25 young with visual impairments (VIs) from a few Educational Centers for Blind Children in Poland. Of these, 21 participants were either blind or had a vision impairment from birth, while the remaining 4 acquired a VI later in life. Participants with acquired visual impairment were represented in all three functional classification groups (B1, B2, and B3), with at least one individual per group.

All surveyed individuals were physically active: boys participated in swimming, goalball, and blind football, while girls engaged in swimming, dancing, and aerobics. The participants were under the legal age (below 18 years of age); thus, their guardians gave written informed consent for them to take part in the study. The participants, after being provided with an explanation of the risks and benefits resulting from participating in this study, as outlined in the Declaration of Helsinki [31], had the option to withdraw from the study at any time. The study was a pilot study within the framework of our own research project “Assessment of the locomotor skills of children and adolescents with visual impairment”, accepted by the Ethics Committee of the University of Physical Education in Warsaw (SKE 01-01/2020 date of approval 22 February 2022) and its ClinicalTrials ID is: NCT06318000.

The inclusion criteria included written informed consent, a clinically confirmed diagnosis of visual impairment, and normative intellectual functioning. The exclusion criteria included the absence of informed consent, any injury that could interfere with task performance, unwillingness to participate, intellectual disability, or lack of a confirmed diagnosis of visual impairment.

Since the study focused on assessing their functional abilities, participants were classified according to the sports classification criteria set by the International Blind Sports Federation (IBSA). It is important to note that the sports classification scale does not determine the cause of the visual impairment but rather assesses its functional relationship with physical activity. Participants with VIs were divided into three groups (1–3) according to the classification of the IBSA: B1—visual acuity poorer than LogMAR 2.60; B2—visual acuity ranging from LogMAR 1.50 to 2.60 and visual field below 10 degrees; and B3—visual acuity ranging from LogMAR 1.40 to 1.0 and visual field below 40 degrees [32] (Table 1).

### 2.2. Procedures

Static balance was assessed using the AMTI AccuSway stabilographic platform (ACS Model). The study participants performed the following tests: standing on both feet with eyes open (BFEO) and closed (BFEC) (30 s), single left- and right-leg stance with eyes open (SLEO and SREO), and single left- and right-leg stance with eyes closed (SLEC and SREC) (10 s). During the tests performed on both feet, the subjects were asked to stand still with their arms at their sides, looking straight ahead. In single-leg stance tests, one leg was raised and bent at a 45-degree angle at the hip, with arms at the sides. The participants were allowed a second attempt in single-leg tests if the first attempt was not successful. The statistical analysis included CoP (center of pressure) path length (cm) and area of stabilogram (cm^2^). Data regarding body height (cm), body mass (kg), age, and visual impairment level were obtained during the study.

Fundamental motor skills related to locomotion were assessed using the Test of Gross Motor Development, Third Edition (TGMD-3), developed by Dr. Dale Ulrich, manufactured in the USA. This tool is an adaptation of the earlier version, TGMD-2, which was developed in part for the assessment of gross motor skills in individuals with visual impairments [29,33]. The TGMD-3 is a standardized and widely validated tool for assessing fundamental motor skills in children and adolescents. It demonstrates high reliability (e.g., inter-rater reliability: 0.98) and strong validity, supported by confirmatory factor analyses and item discrimination metrics. Its applicability has also been confirmed for children and adolescents with visual impairments, showing strong internal consistency and inter-rater agreement—a conclusion repeatedly emphasized across various studies referenced throughout this manuscript [27,33,34,35,36].

The assessment consists of 12 components: 6 focusing on locomotor skills and 6 on object control skills. The locomotor component includes the following six skills: running, galloping, hopping, skipping, jumping, and sliding. Prior to each trial, participants were thoroughly familiarized with the specific motor skill and given the opportunity to perform a practice trial before the actual scored trial.

The only adaptation was the inclusion of an auditory guide positioned at the end of the test path, who provided verbal cues to help participants orient themselves in the correct direction. The demonstration was carried out using tactile methods used in teaching movement to people with visual impairments. A trial performance of the test was carried out to ensure that the child knew how to perform it. Each task was performed twice, with 1 point awarded for correct execution and 0 points for incorrect execution according to predefined criteria. The total score from both trials gave the final score for each task [35].

In the following trials—running, galloping, horizontal jump, and sliding—a maximum of 8 points could be obtained per skill; the hopping allowed for a maximum of 10 points, and the skipping for 6 points. The final score was the sum of points awarded for each task, assessed jointly by three experts in accordance with the guidelines and scoring criteria outlined in the test manual [27,30,37]. The maximum attainable raw score for the “Locomotor” subtest was 48 points. The research results confirm its usefulness in assessing the level of motor skills in the field of locomotion in individuals with visual impairments [36].

### 2.3. Statistical Analysis

SPSS Statistica software version 13.0 (TIBCO Software Inc. Santa Clara, CA, USA) was used to conduct the statistical analysis. For all the analyses, the level of statistical significance was set at *p* < 0.05.

The Shapiro–Wilk test was used to analyze the normal distribution of variables. Levene’s test was used to analyze the homogeneity of variance. The differences in balance levels and motor abilities in locomotion with regard to the degree of impairment, sex, and test conditions (eyes open and eyes closed) were established with the use of the following tests: ANOVA, ANOVA for repeated measures, Kruskal–Wallis’, Friedman’s, Dunn’s, and Wilcoxon’s tests. For the ANOVA, the post hoc test was Tukey’s test; for the Kruskal–Wallis test, it was Dunn’s test; and for the non-parametric Friedman test, it was the Wilcoxon test. The interpretation of the effect size for the ANOVA and Kruskal–Wallis test was as follows: small effect: 0.01 < η^2^ < 0.06; moderate effect: 0.06 < η^2^ < 0.14; large effect: η^2^ ≥ 0.14. For between test conditions, Hedges’ g = 0.20, 0.50, and 0.80, respectively. The relationships between balance levels, age, and the score in the locomotion subtest (TGMD-3) in the groups were established with the use of Pearson’s correlation. The power of relationships was established at the following levels: trivial or none (0.01–0.09), low to medium (0.10–0.29), medium to essential (0.30–0.49), essential to very strong (0.50–0.69), very strong (0.70–0.89), and almost perfect (0.90–0.99 [38]).

## 3. Results

The conducted tests and data analysis revealed significant differences in balance levels between subjects from groups B1 and B2 in tests on the left leg with eyes open on the basis of the surface area of the stabilogram (Kruskal–Wallis, *p* = 0.015) and the CoP path length (Kruskal–Wallis, *p* = 0.046). Subjects with less serious impairment (B3) had better balance levels. Individuals from groups B1 and B2 were different with regard to balance levels on the basis of the surface area of the stabilogram in tests on the left leg with eyes open (Kruskal–Wallis, *p* = 0.04). The degree of impairment among the studied individuals did not differ in terms of motor skills in locomotion (Table 2).

We did not find significant differences in balance levels with eyes open and eyes closed in any of the studied groups (B1, B2, B3) in double-leg stance trials. The conditions in which the trials were conducted (eyes open and eyes closed) differentiated between the balance levels of B2 group individuals in tests both on the right leg and left leg and B3 group individuals on the right leg (Table 3). The studied individuals had better results in trials with eyes open (Table 2).

We observed significant differences in balance performance between eyes-open and eyes-closed conditions in boys during double-leg stance trials (F = 7.76, *p* = 0.01), left-leg stance trials (F = 4.76, *p* = 0.042), and right-leg stance trials (F = 23.6, *p* = 0.001). Girls had significantly better balance levels based on the path length of the stabilogram in comparison to boys in trials with eyes closed on the left leg 68.21 ± 19.63 vs. 99.64 ± 25.30; *p* = 0.03) and right leg (67.48 ± 16.41 vs. 100.09 ± 25.83; *p* = 0.008).

We found significant differences between balance and the level of motor abilities in locomotion in B1 group individuals (double-leg stance trials with eyes closed) and B2 group individuals (single left-leg stance trials with eyes open and eyes closed) (Table 4).

## 4. Discussion

The aim of this study was to assess balance level and fundamental motor skills in the locomotion of youth with regard to their level of visual impairment. Its relevance stems from the fact that visual impairment limits interaction with the environment, often leading to postural instability and difficulties in locomotion [2]. Our findings indicate significant differences in balance performance between individuals with the most profound (B1) and mildest (B3) levels of impairment. Interestingly, B1 individuals demonstrated overall worse balance performance, particularly in static conditions (Table 2). The study results confirm this. Individuals with more serious visual impairment have poorer results in comparison to their peers with a less serious degree of impairment or without visual impairment, particularly in terms of static balance.

Previous studies have shown that visual information plays an important role in maintaining a stable position [23,39]. A limited ability to receive visual stimuli results in distorted postural control. The degree of impairment significantly impacts balance on stable surfaces, and on unstable ones, the testing conditions (eyes open vs. closed) also matter [40]. Our results showed that in double-leg stance trials, these visual conditions did not significantly affect any of the groups (B1–B3). However, in single-leg stance trials, we found differences between eyes open and closed conditions in groups B2 and B3 (Table 3). In particular, the difference between B2 and B3 in the single-leg stance with eyes closed showed an essential to very strong (EVS to VS) effect size, confirming the practical relevance of the observed performance gap. This aligns with findings by Choi et al. [41], who reported that individuals with visual impairment perform better with eyes open. Our study did not confirm such differences in the B1 group, likely because participants in this group could not rely on visual input at all.

When analyzing by sex, we observed no significant differences in balance among girls under different visual conditions in the single-leg stance. Among boys, however, there were noticeable differences, potentially due to differences in the type of physical activities they engage in. Activities such as dance and aerobics, which were more common among girls, involve frequent single-leg stances and movements requiring postural control without relying on visual cues. Additionally, girls in adolescence are often reported to have better coordination and balance, whereas boys may excel in strength and endurance [42].

Postural instability in individuals with visual impairment can affect gait mechanics, often leading to wider steps, bent knees, slower walking speed, and longer double-support phases [4,11,23]. These gait abnormalities increase the risk of falls and suggest broader challenges in developing fundamental motor skills, especially in locomotion [43,44]. Although previous research indicates delayed acquisition of such skills among children with visual impairment [10,23], our results did not confirm these findings. This discrepancy may stem from the high physical activity levels of the participants in our study. Physical activity is a known compensatory factor in motor development [28,45].

The average locomotion score in our sample was 45.4 ± 1.76 out of a maximum of 48, with no significant differences between boys and girls. These results support previous studies suggesting comparable locomotion abilities across sexes in visually impaired youth [13,28,46]. Although the degree of impairment did not significantly affect overall locomotion scores, group B3 achieved the highest values (Table 2), which contradicts earlier findings by Haibach et al. [28] and Uysal and Duger [6]. This may be attributed to the participants’ high levels of physical activity, which could have mitigated typical deficits.

Numerous studies confirm that active children with visual impairment show better motor performance than their inactive peers [9,17,18,26,47,48]. Physical inactivity is associated with poor coordination, inefficient running patterns, and difficulties with phase transitions in movements. One possible reason for these difficulties is the limited opportunity to observe and imitate others due to the lack of visual feedback [23]. Overall, our findings highlight the importance of general physical activity in supporting the development of fundamental motor skills in children with visual impairments [49].

We also identified notable relationships between balance performance and locomotor skill levels, particularly in the B2 and B3 groups (Table 4). For B2 participants, performance in single-leg balance on the non-dominant (left) leg was associated with better locomotor skills, suggesting that targeted training may support development in this area. In group B1, we observed a non-significant trend of decreasing locomotor performance with age (R^2^ = –0.57, *p* = 0.23), while in B3 participants, locomotor skills appeared to increase with age (R^2^ = 0.68, *p* = 0.21). These opposing trends suggest a need for age-specific strategies to support development across impairment levels.

## 5. Limitations and Recommendations to the Future Studies

This research represents one of the first studies in Poland, and is among a few in Europe, to examine balance and locomotor skills in youth with varying degrees of visual impairment. However, some methodological limitations must be acknowledged. The participant pool was limited to children attending a specific sports camp, which resulted in a relatively small and selective sample. Furthermore, this study was conducted as part of a pilot project (“Assessment of the locomotor skills of children and adolescents with visual impairment”), which limited the breadth of its conclusions.

Although participants were relatively homogenous in age, body composition, and physical activity levels, more precise assessment of physical activity (e.g., frequency, duration, and intensity) would provide deeper insights. Our study classified participants as “physically active” based on their involvement in additional sports activities, without further specification.

Another limitation concerns the lack of detailed information regarding the onset of visual impairment in participants with an acquired VI. Future studies should not only expand the sample size but also include participants who are physically inactive and consider a more precise temporal classification of visual impairment onset to better understand its potential impact on motor development and functional abilities.

## 6. Conclusions

These conclusions offer a direct response to the research questions posed and provide both theoretical and practical implications for supporting motor development in youth with visual impairment.

The findings of this study underscore the importance of balance in the development of motor skills among youth with visual impairments. Fundamental motor skills are crucial for engaging in physical activities, games, and daily tasks, contributing to overall functional independence. These skills are also essential for preventing lifestyle-related diseases by promoting active leisure.

Although our sample included only physically active individuals, the results offer valuable cognitive and practical conclusions:

1. Higher balance levels (eyes closed, single-leg stance) in girls than boys may be attributed to sex-based differences in motor abilities during adolescence.
2. The lack of differences in girls’ trials with eyes open vs. closed may result from their involvement in activities like dance and fitness, supporting their inclusion in physical education curricula.
3. Differences in balance across groups with varying degrees of visual impairment highlight the impact of sensory loss on balance development.
4. The absence of significant sex differences in locomotor skills suggests equal potential for motor development in boys and girls with visual impairment.
5. High locomotion scores emphasize the benefits of regular physical activity and suggest that visual impairment need not be a barrier to motor skill acquisition.
6. The observed associations between balance and locomotion support the need for targeted interventions that enhance postural control.
7. The age-related increase in motor skills among those with milder impairment and the decline among those with severe impairment highlight the importance of early and tailored interventions.

## Figures and Tables

**Table 1:** Description of the study group (x ± SD).

	Boys (*n* = 15)			Girls (*n* = 10)		
Height [cm]	166.9 ± 13.1			166.0 ± 12.6		
Body mass [kg]	57.3 ± 13.3			57.4 ± 13.1		
Age [years]	15.2 ± 1.6			15.2 ± 1.6		
Visual impairment level	B1	B2	B3	B1	B2	B3
	20%	60%	20%	30%	50%	20%

**Table 2:** The differences in balance levels and motor skills in the locomotion of the studied individuals with regard to the degree of impairment (analysis of variance, ANOVA).

	B1 (*n* = 6) x ± SD	B2 (*n* = 14) x ± SD	B3 (*n* = 5) x ± SD	F or H	*p*	ES *η*^2^
BF EO Area Circ [cm^2^]	2.30 ± 0.89	2.38 ± 1.05	1.85 ± 0.52	0.69	0.518	0.05
BF EO Path Lenght [cm]	48.1 ± 3.52	39.9 ± 10.1	39.2 ± 12.3	1.12	0.352	0.14
BF EC Area Circ [cm^2^]	2.35 ± 1.06	3.02 ± 2.3	2.05 ± 0.55	0.41	0.671	0.05
BF EC Path Lenght [cm]	47.7 ± 11.3	45.1 ± 15.0	48.6 ± 13.2	0.72	0.502	0.01
SL EO Area Circ [cm^2^]	24.0 ± 11.6	9.88 ± 3.54 *	7.42 ± 1.70 *	**8.81 ^#^**	**0.011**	**0.34**
SL EO Path Length [cm]	90.6 ± 30.8	62.1 ± 14.8	54.0 ± 4.72 *	**6.02 ^#^**	**0.049**	**0.20**
SL EC Area Circ [cm^2^]	19.2 ± 10.1	21.2 ± 9.28	15.6 ± 5.26	0,89	0.431	0.07
SL EC Path Lenght [cm]	93.6 ± 31.2	78.8 ± 25.0	88.6 ± 31.8	0.76	0.485	0.06
SR EO Area Circ [cm^2^]	14.1 ± 8.00	14.4 ± 10.9	9.74 ± 2.76	2.40	0.123	0.04
SR EO Path Lenght [cm]	74.3 ± 24.9	67.0 ± 16.5	66.0 ± 20.2	2.97	0.080	0.03
SR EC Area Circ [cm^2^]	19.1 ± 8.97	17.0 ± 5.7	21.1 ± 4.61	1.88	0.185	0.09
SR EC Path Lenght [cm]	89.8 ± 31.2	80.8 ± 28.0	95.9 ± 25.2	2.83	0.089	0.06
TGMD-3 (locomotion) (points)	44.2 ± 1.33	45.7 ± 1.73	46.0 ± 1.87	1.08	0.362	0.17

B1, B3, and B3—visual impairment level; BF—both feet; SL—single left; SR—single right; EO—eyes open; EC—eyes closed. *—statistically different from B1; ^#^—Kruskal–Wallis H statistic.

**Table 3:** Differences in balance levels with eyes open and eyes closed with regard to the degree of impairment, based on the path length of the stabilogram.

		*n* Valid	T	Z	*p*	ES Hedges’ *g*
EO vs. EC (L)	B1	5	5.00	0.67	0.50	−0.09
EO vs. EC (R)		4	3.00	0.73	0.46	−0.54
EO vs. EC (L)	B2	11	10.00	**2.04**	**0.04**	**−0.77**
EO vs. EC (R)		12	5.00	**2.66**	**0.00**	**−0.59**
EO vs. EC (L)	B3	5	1.00	1.75	0.07	−1.20
EO vs. EC (R)		5	0.00	**2.02**	**0.04**	**−1.09**

B1, B2, and B3—visual impairment level; EO—eyes open; EC—eyes closed; R—right; L—left.

**Table 4:** Balance levels on the basis of tests with eyes open and eyes closed and level of motor abilities in locomotion with regard to the degree of impairment of the studied individuals.

	B1 (*n* = 6)				B2 (*n* = 14)				B3 (*n* = 5)			
	r	t	*p*	ES	r	t	*p*	ES	r	t	*p*	ES
BF EO Area Circ	−0.61	−1.54	0.20	EVS	−0.27	−0.98	0.35	LM	0.24	0.43	0.70	LM
BF EO Path Lenght	0.51	1.19	0.30	EVS	−0.12	−0.43	0.67	LM	−0.06	−0.11	0.92	TN
BF EC Area Circ	0.68	1.87	0.13	EVS	−0.07	−0.26	0.80	TN	0.19	0.35	0.75	LM
BF EC Path Lenght	**0.93**	**5.09**	**0.007**	AP	−0.09	−0.32	0.76	TN	0.15	0.27	0.81	LM
SL EO Area Circ	0.52	1.07	0.36	EVS	**−0.56**	**−2.23**	**0.05**	EVS	−0.29	−0.53	0.63	LM
SL EO Path Length	0.68	1.60	0.21	EVS	−0.13	−0.42	0.68	LM	−0.37	−0.70	0.53	ME
SL EC Area Circ	0.30	0.54	0.62	ME	**0.65**	**2.60**	**0.03**	EVS	0.27	0.48	0.66	LM
SL EC Path Lenght	0.67	1.58	0.21	EVS	**0.67**	**2.71**	**0.02**	EVS	0.26	0.46	0.67	LM
SR EO Area Circ	0.28	0.58	0.59	LM	−0.13	−0.46	0.66	EVS	0.68	1.62	0.20	EVS
SR EO Path Lenght	0.53	1.25	0.28	EVS	−0.05	−0.19	0.85	TN	0.56	1.19	0.32	EVS
SR EC Area Circ	0.91	3.15	0.09	AP	−0.11	−0.35	0.73	LM	0.01	0.02	0.98	TN
SR EC Path Lenght	0.76	1.63	0.24	VS	0.10	0.33	0.74	LM	0.47	0.91	0.43	ME

B1, B3, and B3—visual impairment level; BF—both feet; SL—single left; SR—single right; EO—eyes open; EC—eyes closed. ES—effect size; AP—almost perfect; VS—very strong; EVS—essential to very strong; ME—medium to essential; LM—low to medium; TN—trivial to none.

## Data Availability

Results of BOT-3 and TGMD-3 Tests in Youth with Vision Dysfunction Database is available at https://www.scidb.cn/en/anonymous/TTc3N2ph, accessed on 4 November 2024 with permission from all the authors to use it for further research.
